# The cholesterol metabolite 25-hydroxycholesterol suppresses porcine deltacoronavirus via lipophagy inhibition and mTORC1 modulation

**DOI:** 10.1186/s13567-025-01452-9

**Published:** 2025-01-31

**Authors:** Jia-lu Zhang, Xue-fei Wang, Jia-lin Li, Cong Duan, Jiu-feng Wang

**Affiliations:** 1 National Key Laboratory of Veterinary Public Health and Safety, College of Veterinary Medicine, China Agricultural University, Beijing, 100193 China; 2 College of Veterinary Medicine, Southwest University, Chongqing, 400715 China; 3 China Institute of Veterinary Drug Control, Beijing, 100081 China

**Keywords:** Porcine coronavirus, 25-Hydroxycholesterol, lipophagy, transcription factor EB, piglets

## Abstract

25-Hydroxycholesterol (25HC) is a hydroxylated cholesterol with multiple antiviral activities, however, little is known about the mechanisms by which 25HC correlates antiviral ability with lipid droplet (LD) dynamic balance to ensure cholesterol homeostasis. In the present study, 25HC was applied to porcine deltacoronavirus (PDCoV)-infected LLC-PK1 (Lilly Laboratories Culture-Porcine Kidney 1) cells and piglets to explore its antiviral capacity and underlying mechanism. The results revealed that 25HC decreased free cholesterol (FC) levels but increased triglyceride (TG) levels in PDCoV-infected cells and piglets. The accumulation of LDs induced by oleic acid (OA) impedes PDCoV replication. In addition, 25HC administration increases LD accumulation and declines protein expression associated with lipophagy and lysosomes to facilitate LD accumulation. Moreover, 25HC inhibited TFEB (transcription factor-EB) expression, blocked its translocation into the nucleus and reversed Mechanistic Target of Rapamycin Complex 1 (mTORC1) activity, which in turn hindered lipophagy and PDCoV replication. Additionally, 25HC treatment ameliorated the clinical symptoms and intestinal injury of PDCoV-infected piglets. These findings reveal the beneficial effect of lipophagy on PDCoV infection and uncover the antiviral mechanism of 25HC, by which lipophagy and mTOR activity are tightly controlled by 25HC.

**Graphical Abstract:**

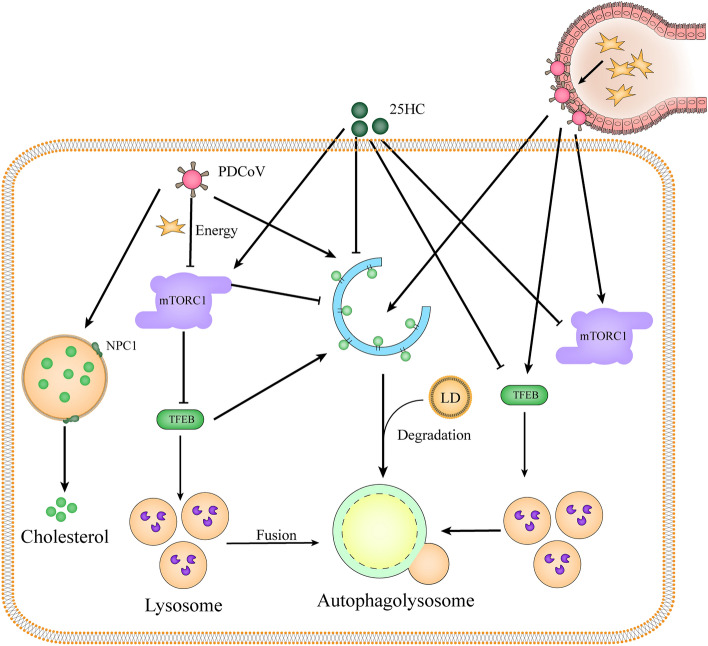

## Introduction

Coronaviruses are highly variable, enveloped RNA viruses that threaten human and livestock health [1]. Porcine Deltacoronavirus (PDCoV) has emerged as a main enteropathogenic coronavirus, causing watery diarrhea in newborn piglets and leading to substantial economic losses in the pig industry [2]. Moreover, because of its smallest genome among coronaviruses, PDCoV exhibits increased prevalence and potential to threaten the health of both animals and humans [3, 4]. Recently, scientists have isolated PDCoV strains from the blood of Haiti children, three of which presented with acute undifferentiated febrile illness [5]. Therefore, efforts to decipher the pathogenic mechanism and cellular response following PDCoV infection can help control the epidemic and serve as a theoretical basis for human coronavirus research.

Cholesterol plays pivotal roles in maintaining cellular function and in the viral lifecycle [6]. It constitutes the viral envelope and facilitates viral membrane fusion by causing disorders of plasma membrane cholesterol [7]. After the initial step, viruses exploit cholesterol metabolism to benefit their replication and assembly [8]. Excess cholesterol in normal cells can be esterized into cholesteryl ester and, consequently, stored in lipid droplets (LDs) [9], highly dynamic organelles involved in various diseases [10] and utilized by some viruses, such as Dengue virus (DENV), to create compartments for viral replication [11]. Interestingly, the phenomenon in which LDs accumulate along with enhanced interferon-β (IFN-β) in the early stages of infection by some viruses also suggests the antiviral potential of LDs [12], indicating the necessity of LD degradation for viral infection. Autophagy, a critical process for recycling proteins and organelles under nutrient insufficiency [13], involves microtubule-associated protein light chain 3-II (LC3-II) and the adaptor protein p62/SQSTM1, which are essential for autophagosome formation and cargo degradation [14]. Lipophagy, a type of selective autophagy, degrades LDs to maintain cellular homeostasis [15].

25-Hydroxycholesterol (25HC), derived from cholesterol hydroxylation catalyzed by cholesterol-25-hydroxylase (CH25H) [16], is recognized as a regulator of cholesterol metabolism [17]. 25HC exhibits antiviral properties against various porcine viruses, including porcine reproductive and respiratory syndrome virus (PRRSV) and PDCoV [18, 19]. Previous studies have demonstrated that early infection with PDCoV is accompanied by LD accumulation at 8 hpi, nevertheless, the application of 25HC further enhances LD quantity and induces the transcription of IFN-β [20]. However, the specific mechanisms by which PDCoV manages accumulated LDs and the underlying anti-PDCoV mechanisms of 25HC require further investigation.

This study aimed to elucidate how PDCoV regulates LDs and the role of 25HC in maintaining cellular homeostasis. By exploring these interactions, including viral exploitation of host lipophagy and the effects of 25HC on lysosomal and autophagic pathways, this research reveals a critical intersection between coronavirus infection and cellular metabolism.

## Materials and methods

### Chemicals

25-Hydroxycholesterol (25HC) (purity: ≥ 98.0%) (HY-113134), bafilomycin A1 (Baf A1) (HY-100558), Torin1 (HY-13003), U18666A (HY-107433), and protease inhibitors (HY-K0010 and HY-K0022) were ordered from MedChemExpress (Beijing, China). Oleic acid (O1383) was obtained from Sigma-Aldrich (Beijing, China).

### Cell culture and virus infection

The LLC-PK1 (Lilly Laboratories Culture-Porcine Kidney 1) cell line (CL-101) was purchased from the American Type Culture Collection (ATCC) and cultured in DMEM medium (Gibco, USA). The media was supplemented with 10% heat-inactivated fetal bovine serum (FBS) (Gibco, USA), 1% antibiotic–antimycotic (Gibco, USA), 1% nonessential amino acid solution (NEAA) (Gibco, USA). The cells were cultured in a humidified incubator at a constant temperature of 37 ℃ under 5% CO_2_.

The PDCoV strain (CHN-HN-1601, GenBank accession no. MG832584) was donated by Professor Hanchun Yang of China Agricultural University. LLC-PK1 cells were infected with PDCoV at MOI of 0.1 for 1 h, and the media was changed. The cells were adequately washed to rid unbounded virus and then cultured in DMEM medium supplemented with 0.4% trypsin (T1350, Solarbio Science & Technology Co. Ltd.) at 37 ℃ for the indicated time.

### Animal experiment

Fifteen piglets (Bama miniature pigs, 7 days old) were purchased from a commercial farm in Tianjin, China, housed in cages at room temperature and supplied with water and food ad libitum. The negative results for porcine enteric viruses including PDCoV, porcine epidemic diarrhea virus (PEDV), transmissible gastroenteritis virus (TGEV) and rotavirus were confirmed before the start of the experiment, and no clinical symptoms were observed. The piglets were divided randomly into three groups: the control group (Ctrl), the PDCoV infection group (PDCoV) and the 25HC treatment group (PDCoV + 25HC). A PDCoV infection model was constructed by oral administration of 1 × 10^6^ TCID_50_ PDCoV strain at day 1. Piglets in the 25HC treatment group were administrated with 25HC (10 mg/kg bw^−1^), while piglets in the PDCoV infection group were given vehicle (HβCD, 10 mg/mL) by intraperitoneal injection after the PDCoV infection from days 1 to 5. On day 6, all the piglets were sacrificed, and tissue and blood samples were collected.

The diarrhea score was evaluated according to previously described criteria [21]: 1, normal feces, no adherence to the ground. 2, normal feces, slightly wet on the surface. 3, soft feces, wet surface. 4, soft feces, very wet. 5, soft feces, semisolid and very wet. 6, watery feces.

### Blood leukocyte counts

Part of the blood was collected in an anticoagulant tube containing EDTA. Blood leukocytes, including lymphocytes, monocytes, neutrophils, eosinophils and basophils, were measured via an automated blood cell analyzer (Sysmex XN-1000 V, Japan).

### ELISA analysis

A portion of the blood was placed in ordinary tubes for the collection of serum. A porcine- IFN-β ELISA kit (JL11792) was purchased from Jianglai Biotechnology Co. Ltd. (Shanghai, China), and a porcine-IgA ELISA kit (SEKP-0013) was purchased from Solarbio Science & Technology Co. Ltd. (Beijing, China). ELISAs were performed according to the manufacture’s protocols.

### Western blot analysis

Cultured cells and tissues were collected and lysed in RIPA buffer supplemented with 1% protease and phosphatase inhibitor cocktail on ice. Nuclear and cytoplasmic proteins were extracted via a nuclear protein and cytoplasmic protein extraction kit (P0027, Beyotime Biotechnology, China) according to the protocol. Proteins were separated by SDS-PAGE and then transferred to 0.45 μm polyvinylidene fluoride (PVDF) membranes (Millipore, USA). After incubation in 5% skim milk (A600669, Sangon Biotech, Shanghai, China) for 2 h at room temperature, the membrane was then incubated with the indicated primary antibodies overnight at 4 ℃. After incubation with horseradish peroxidase-conjugated secondary antibody for 1 h at room temperature, the protein bands were detected with an enhanced chemiluminescence (ECL) detection system (Tanon 6200 imaging workstation, Tanon Science & Technology, China) and quantified via Image J. The primary antibodies used are shown in Table 1.

**Table 1:** **Antibodies used for western blot**

Antibody	Product code	Manufacturer	Dilution
LC3	14600-1-AP	Proteintech	1:2000
Beclin1	11306-1-AP	Proteintech	1:1000
ATG5	10181-2-AP	Proteintech	1:1000
p62	18420-1-AP	Proteintech	1:1000
LIPA	12956-1-AP	Proteintech	1:1000
PLIN2	15294-1-AP	Proteintech	1:1000
LAMP1	21997-1-AP	Proteintech	1:10 000
Cathepsin D	55021-1-AP	Proteintech	1:3000
ATGL	55190-1-AP	Proteintech	1:500
TFEB	13372-1-AP	Proteintech	1:2000
NPC1	13926-1-AP	Proteintech	1:500
SREBP2	28212-1-AP	Proteintech	1:2000
P70S6K	14485-1-AP	Proteintech	1:2000
p-P70S6K	28735-1-AP	Proteintech	1:2000
GAPDH	60004-1-Ig	Proteintech	1:5000
β-actin	60008-1-AP	Proteintech	1:5000
Tubulin	10068-1-AP	Proteintech	1:5000
Histone-H3	17168-1-AP	Proteintech	1:10 000
PDCoV-N	SD-4-5	Medgene Labs	1:500
4EBP1	#9452	Cell Signaling Technology	1:1000
p-4EBP1	#2855	Cell Signaling Technology	1:1000
mTOR	T55306	Abmart	1:1000
Phosphorylation-mTOR	ab109268	ABCAM	1:1000

### qRT-PCR analysis

Total RNA from cells and tissues was isolated using RNAiso Plus (9109, Takara, Japan), and cDNA was then synthesized by using TransStart one-step gDNA removal and cDNA synthesis supermix kit (AT311, TransGen Biotech, China) according to the protocols. The amplification was performed in triplicate using TransStart qPCR SuperMix (AQ132, TransGen Biotech, China). The housekeeping gene GAPDH was used as an internal control, and the results were normalized against GAPDH and were quantified via the comparative CT method (2^–ΔΔCt^). The primers used are shown in Table 2.

**Table 2:** **Primers used for qPCR**

Gene	Direction	Sequence (5′ → 3′)	Production Size
PDCoV N	Forward	AACTTTCAGGCAGGGGCAAT	134 bp
	Reverse	GGTTTGGTGGGTGGCTCATA	
GAPDH	Forward	ACATGGCCTCCAAGGAGTAAGA	106 bp
	Reverse	GATCGAGTTGGGGCTGTGACT	
IFN-β	Forward	TTCGAGGTCCCTGAGGAGATT	176 bp
	Reverse	TCCATCTGCCCATCAAGTTCC	
TFEB	Forward	GGACACCTGAACTTGGGACAA	83 bp
	Reverse	TGCTGGCTCCCTATCTCTGA	
NPC1	Forward	GCTACCGTGTGTTCCCCTAC	92 bp
	Reverse	GGACACGCCGAGGTTAAAGA	
MX1	Forward	GTGGAGAAAAGTCACAAAACAGGGC	288 bp
	Reverse	TTTGCCCTTCCATTCGTCTTCT	
ISG15	Forward	GGTGAGGAACGACAAGGGTC	177 bp
	Reverse	GGCTTGAGGTCATACTCCCC	
ISG20	Forward	CAGGACAGTGTGTTGGGACA	101 bp
	Reverse	ACAGGCAGCCCTCATTGTTC	

### Histological and immunofluorescence staining

The intestine samples were isolated and fixed in 4% paraformaldehyde. After dehydration and embedding in paraffin, histological section (3 μm) were acquired and stained with hematoxylin and eosin (H&E). The images were captured via light microscopy (Olympus IX71, Japan). The histopathological score was evaluated according to previous criteria [21].

The cells were cultivated on glass coverslips in 24-well cell plates, and the tissue samples were made into paraffin sections. After that, the cells and paraffin sections were fixed in 4% paraformaldehyde (PFA) for 15 min at room temperature. After permeabilization with 1% Triton X-100 in PBS for 10 min, the cells were blocked with 2% BSA for 1 h at room temperature. The sections were incubated with anti-PDCoV-N, and the cells were incubated with anti-TFEB, anti-LC3, anti-LAMP1, and anti-NPC1 antibodies overnight at 4 ℃. The secondary antibodies Alexa Fluor-488 (A-11008, Thermo Fisher Scientific, USA), Alexa Fluor-647 or Alexa Fluor-555 (A0468 and A0460, Beyotime Biotechnology Co. Ltd., China) were added for 1 h. DAPI was finally applied to stain the cell nuclei. Images were captured with a confocal laser scanning microscope (Nikon A1, USA).

### BODIPY staining

After fixation in 4% paraformaldehyde for 15 min, the cells were incubated with 2 μM BODIPY 493/503 (1:2500 in PBS, GC42959, GLPBIO, USA) at 37 ℃ in the dark for 15 min and finally stained with DAPI. The images were visualized via a Nikon fluorescence microscope.

### Filipin staining

For filipin staining, the cells were fixed in 4% PFA and then stained with 50 μg/mL filipin III (SAE0088, Sigma-Aldrich, USA) for 2 h at room temperature in the dark. The images were captured via a Nikon fluorescence microscope.

### FC and TG measurement

The cells were cultivated in 10 cm dishes for measurement of metabolites after they reached 90% confluence. For cholesterol measurement, the cells were harvested with isopropanol and sonicated. After centrifugation, the supernatant was collected to measure the content of free cholesterol and total cholesterol via a Free Cholesterol Levels Assay Kit and Total Cholesterol Levels Assay Kit (BC1890 and BC1980, Beijing Solarbio Science & Technology, China) according to the manufacturer’s instructions. For triglyceride analysis, cells were collected and n-Heptane: isopropanol (1:1) was added, after sonication, the supernatant was collected to determine the triglyceride content using a Triglyceride Levels Assay Kit (BC0620, Beijing Solarbio Science & Technology, China) according to the protocol.

### Transfection of siRNA

The cells were grown to 50–70% confluence in serum-free Opti-MEM medium (Gibco, USA) for transfection, and siRNA transfection was performed via RNAimax (13778100, Invitrogen, USA) according to the manufacturer’s instructions. Specific siRNAs for TFEB and NPC1 were obtained from GenePharma (Suzhou, China). The siRNA sequences for specific genes are shown in Table 3.

**Table 3:** **Sequences of siRNAs**

Gene	Direction	Sequence (5′ → 3′)
siTFEB	Sense	GCGACAGAAGAAGGACAAUTT
	Antisense	AUUGUCCUUCUUCUGUCGCTT
siNPC1	Sense	GCAUGUUCCUGUCAUCCUUTT
	Antisense	AAGGAUGACAGGAACAUGCTT

### Transmission electron microscopy

Tissue samples from the jejunum and ileum were collected, cut into 1 mm pieces and then fixed in electron microscopy fixative (2.5% glutaraldehyde, pH 7.4) for 48 h at 4 ℃. The samples were then postfixed in 2.0% osmium tetroxide for 1 h and dehydrated within a graded ethanol series, embedded in Electron Microscopy Sciences (EMbed-812, Fort Washington, PA, USA), sliced into ultrathin sections. The ultrathin sections were stained with uranyl acetate and lead citrate and then observed with an H7500 transmission electron microscope (Hitachi, Tokyo, Japan).

### Statistical analysis

Image production and statistical analysis were performed via GraphPad Prism 9, the data were obtained from at least three independent experiments and are expressed as mean ± standard deviation (SD). Differences were analyzed with Student’s *t* test (unpaired) or one-way analysis of variance (ANOVA) followed by Tukey’s test. *P* values of < 0.05 were considered statistically significant, *P < 0.05, **P < 0.01, ***P < 0.001.

## Results

### 25HC induces LD accumulation and decreases cholesterol abundance

**Figure 1:**
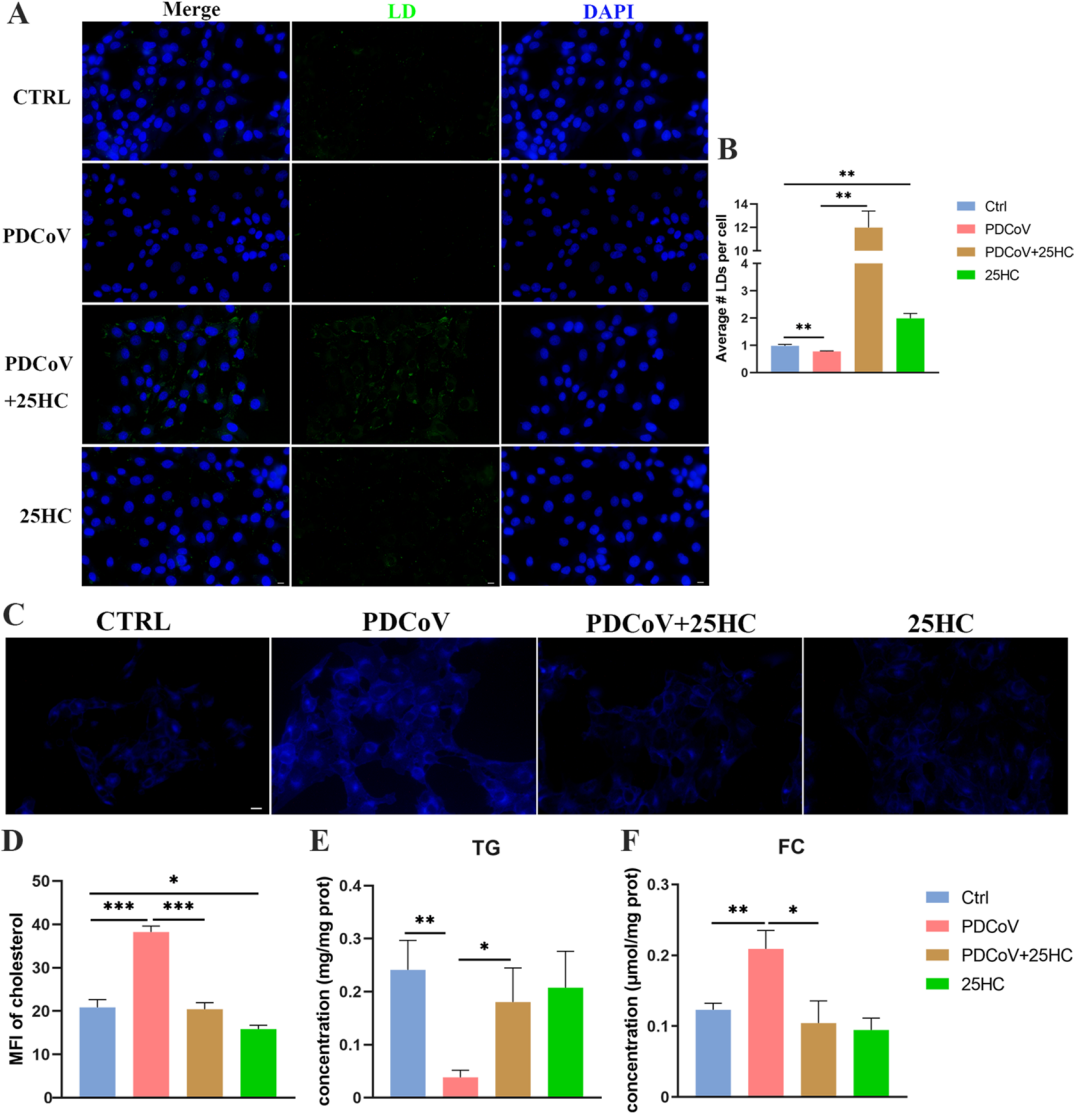
25HC induces LDs accumulation and inhibits cholesterol abundance. **A** LLC-PK1 cells were infected with PDCoV (MOI = 0.1) and treated with 50 μM 25HC for 12 h. Lipid droplets (LD) were stained green with BODIPY (493/503), and nuclei were stained blue with DAPI. Scale bar, 10 μm. **B** Average number of LDs per cell was measured using Image J. **C** Free cholesterol in cells was stained blue with Filipin III. Scale bar, 10 μm. **D** The mean fluorescence intensity (MFI) of cholesterol in panel **C** was analyzed using Image J. **E, F** The cellular concentrations of Triglyceride (TG) and Free cholesterol (FC) were measured in LLC-PK1 cells infected with PDCoV and treated with 25HC for 12 h. Error bars, mean ± SD. *P < 0.05, **P < 0.01, ***P < 0.001.

LD accumulation was increased during early PDCoV infection at 8 hpi compared with that in normal cells [20], suggesting a potential cellular protective response, which might be weakened by prolonged infection. We conducted LD staining in LLC-PK1 cells after PDCoV infection at 12 hpi, and detected a significant decrease in the number of LDs, whereas the application of 25HC dramatically enhanced LD accumulation compared with that in the PDCoV group (Figures 1A and B). In addition, PDCoV infection significantly elevated the level of cholesterol, which was reversed by treatment with 25HC (Figures 1C and D). Further analysis of triglyceride (TG) and free cholesterol (FC) levels confirmed a decrease in TG and an increase in FC following PDCoV infection, both of which were restored to normal levels after the addition of 25HC (Figures 1E and F).

### PDCoV induces active lipophagy in LLC-PK1 cells

**Figure 2:**
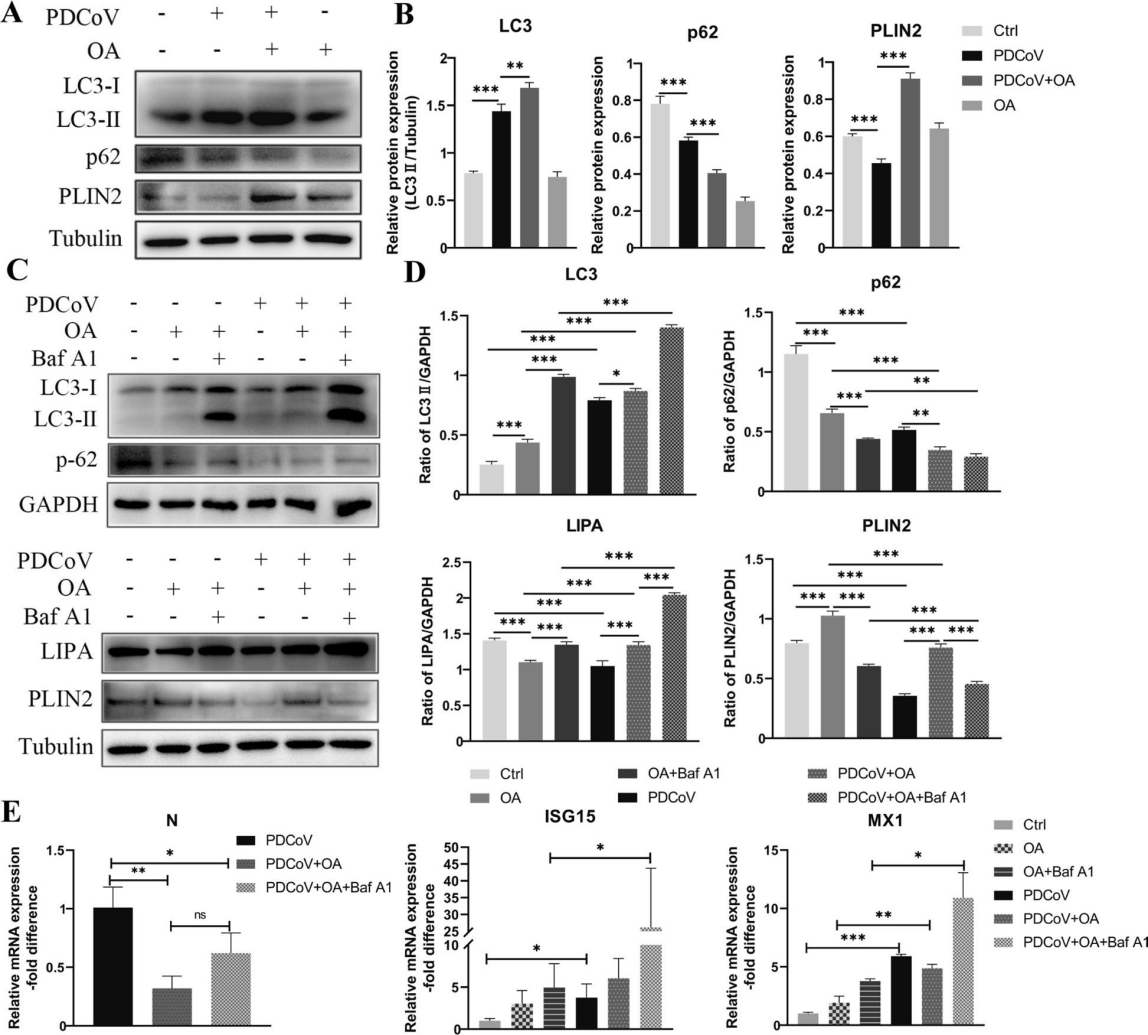
PDCoV induces active lipophagy in LLC-PK1 cells. **A, B** LLC-PK1 cells were infected with PDCoV (MOI = 0.1) and treated with 200 μM Oleic acid (OA) for 12 h. **A** LC3, p62 and PLIN2 expression were analyzed by western blot, Tubulin was used as a loading control. **B** The immunoblot bands in panel A were quantified by Image J. **C–E** LLC-PK1 cells were infected with PDCoV (MOI = 0.1) and treated with 200 μM Oleic acid (OA), 200 nM Bafilomycin A1 (Baf A1) for 12 h. **C** The indicated protein expression were determined by western blot analysis, GAPDH and Tubulin were used as loading control. **D** The immunoblot bands in panel C were quantified by Image J. **E** The relative mRNA expression of PDCoV N, ISG15 and MX1 were measured using qPCR analysis. Error bars, mean ± SD. *P < 0.05, **P < 0.01, ***P < 0.001, ns represents not significant.

PLIN2, a key LD-associated protein belonging to the perilipin protein family, serves as a marker of LDs [22]. Oleic acid (OA) was used to induce the production of LDs, the treatment of OA induced abundant expression of PLIN2 and further promoted the expression of LC3-II and suppressed p62 expression in PDCoV-infected cells. PDCoV infection decreased the levels of PLIN2 and p62, besides, PDCoV induced LC3-II expression, indicating enhanced lipophagy during PDCoV infection. (Figures 2A and B).

To further assess the level of autophagy, we applied bafilomycin A1 (Baf A1) to block the degradation of autophagosomes. PDCoV-induced LC3-II expression was aggravated by Baf A1 treatment. LIPA, an important enzyme for acid lipolysis and lipophagy, was significantly lower at 12 hpi in the PDCoV group than in the control group, whereas its expression increased with the addition of OA and Baf A1. Notably, PDCoV inhibited the expression of PLIN2, which was not restored by the addition of Baf A1 (Figures 2C and D). The addition of OA significantly restricted the replication of PDCoV, as indicated by reduced PDCoV N levels. PDCoV infection increased the expression of ISG15 (Interferon Stimulated Gene 15) and MX1, which further increased after the simultaneous addition of OA and Baf A1 (Figure 2E).

### 25HC inhibits constant lipophagy induced by PDCoV infection

**Figure 3:**
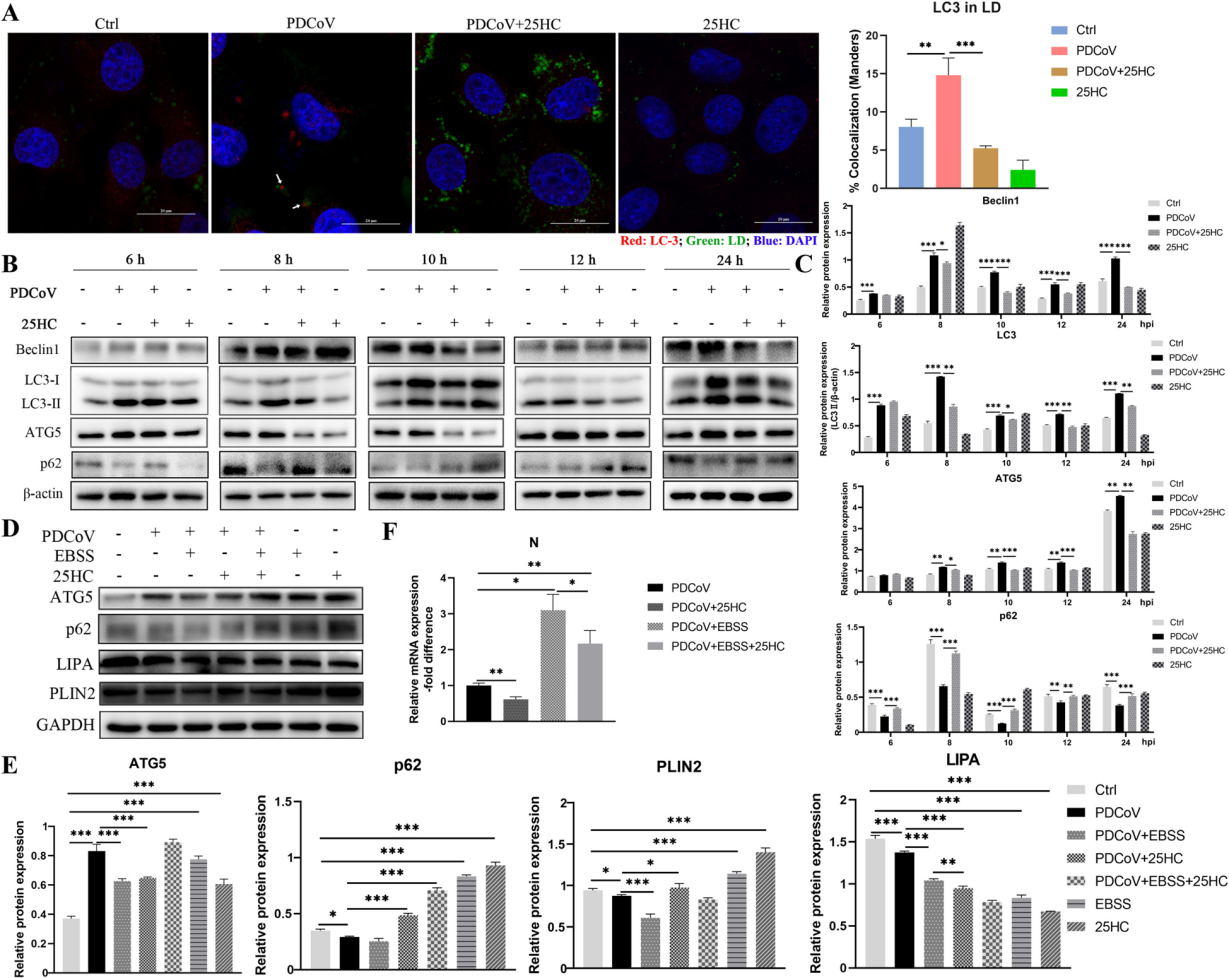
25HC inhibits constant lipophagy induced by PDCoV infection. **A–C** LLC-PK1 cells were infected with PDCoV (MOI = 0.1) and treated with 50 μM 25HC for indicated time. **A** The cells were processed at 12 hpi. LC3 was stained red, LD was stained green and nuclei were stained blue. Scale bar, 20 µm. LC3 recruitment to LDs was quantified by Fiji-Image J. **B** The protein expression of Beclin1, LC3, ATG5 and p62 were analyzed by western blot, β-actin was used as a loading control. **C** The immunoblot bands in panel A were quantified by Image J. **D–F** LLC-PK1 cells were incubated with EBSS for 2.5 h, followed by infection of PDCoV and treatment with 25HC for 12 h. **D** ATG5, p62, LIPA and PLIN2 expression were analyzed by western blot, using GAPDH as a loading control. **E** Relative quantification of bands in panel **D** were conducted by Image J. **F** Relative mRNA expression of PDCoV N was determined by qPCR analysis. Error bars, mean ± SD. *P < 0.05, **P < 0.01, ***P < 0.001.

The colocalization of LC3-II and LDs was increased in PDCoV-infected cells, verifying upregulated lipophagy (Figure 3A). Considering that lipophagy is a dynamic process, sequential time points were chosen to monitor associated protein levels during PDCoV infection and 25HC administration. PDCoV induced constant upregulated expressions of Beclin1, LC3-II and ATG5 and decreased the expression of p62, suggesting that PDCoV activated autophagy flux. The abnormal expressions of Beclin1, ATG5, LC3-II and p62 were reversed by the administration of 25HC (Figures 3B and C).

Earle’s balanced salt solution (EBSS) is an inducer of autophagy that causes a starvation environment. Compared with the PDCoV group, individual addition of EBSS significantly increased the expression of ATG5, whereas EBSS treatment significantly downregulated PLIN2 protein expression in PDCoV-infected cells. Both the EBSS and 25HC treatments decreased LIPA expression after PDCoV infection. The expression of PLIN2 was significantly promoted by the addition of 25HC (Figures 3D and E). EBSS addition to PDCoV-challenged cells significantly promoted viral replication, which was attenuated by 25HC (Figure 3F). Collectively, these results suggest that PDCoV induces constant lipophagy to reduce the LD content, which can be restrained by 25HC treatment.

### 25HC restrains active lysosomal function induced by PDCoV infection

**Figure 4:**
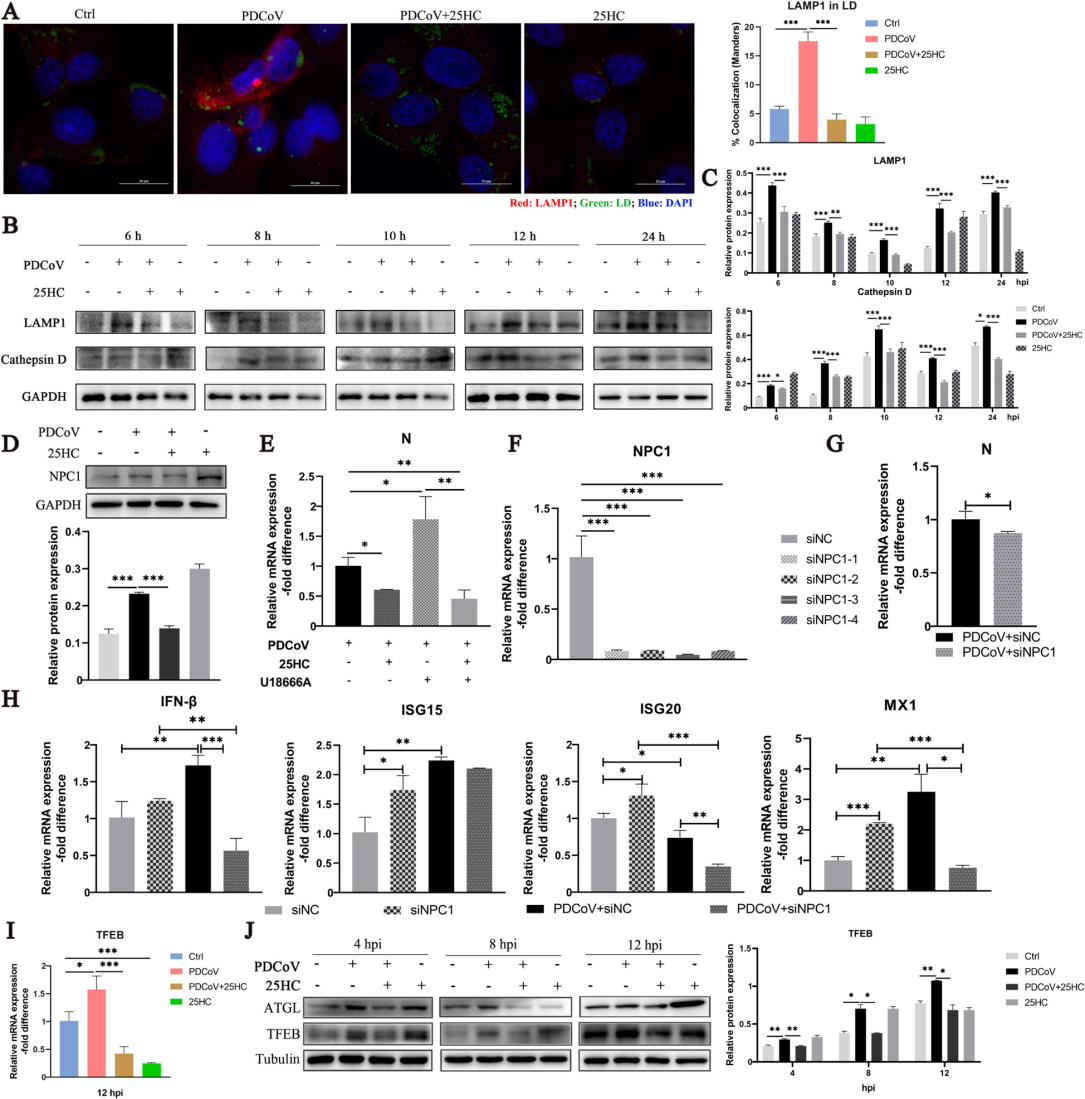
25HC restrains active lysosomal function induced by PDCoV infection. **A–C** LLC-PK1 cells were infected with PDCoV (MOI = 0.1) and treated with 50 μM 25HC for indicated time. **A** The cells were processed at 12 hpi. LAMP1 was stained red, LD was stained green and nuclei were stained blue. Scale bar, 20 µm. LAMP1 recruitment to LDs was quantified by Fiji-Image J. **B** The protein expression of LAMP1 and Cathepsin D were analyzed by western blot, GAPDH was used as a loading control. **C** The immunoblot bands in panel B were quantified by Image J. **D** NPC1 expression was analyzed by western blot, with GAPDH being a loading control, the relative quantification was conducted by Image J. **E** LLC-PK1 cells were infected by PDCoV (MOI = 0.1) and treated by 50 μM 25HC, 10 μM U18666A for 12 h. Relative mRNA expression of PDCoV N was measured by qPCR. **F** LLC-PK1 cells were transfected with four siRNAs targeting NPC1, the relative mRNA expression of NPC1 was analyzed by qPCR. **G, H** LLC-PK1 cells were transfected with the third siRNA in panel F, followed by infection with PDCoV for 12 h. Relative expression of (**G**) PDCoV N and (**H**) IFN-β, ISG15, ISG20 and MX1 were determined by qPCR analysis. **I** Relative mRNA expression of TFEB was measured by qPCR. **J** ATGL and TFEB expression were analyzed by western blot, Tubulin was used as a loading control. Immunoblot bands were quantified by Image J. Error bars, mean ± SD. *P < 0.05, **P < 0.01, ***P < 0.001.

LD degradation in lipophagy involves delivery to lysosomes, hence, we examined the expression of the lysosomal markers LAMP1 and Cathepsin D. The immunofluorescence results revealed increased colocalization of LAMP1 and LDs after PDCoV infection (Figure 4A). In addition, the protein expression levels of LAMP1 and Cathepsin D were obviously increased in PDCoV-infected cells, which was reversed by 25HC administration (Figures 4B and C). NPC1, a protein that resides in the lysosomal membrane, mediates cholesterol trafficking in the lysosome [23]. NPC1 exhibited an expression pattern similar to that of LAMP1 after PDCoV infection and 25HC application (Figure 4D). Next, U18666A, an inhibitor of cholesterol trafficking, was used to assess the effect of NPC1 expression on PDCoV replication, however, the addition of U18666A to PDCoV-infected cells promoted PDCoV N expression (Figure 4E). Considering that U18666A can block the esterification of cholesterol [24], specific siRNAs were designed to interfere with the expression of NPC1 (Figure 4F), and siNPC1-3 was selected for subsequent experiments. Notably, the expression of PDCoV N was significantly reduced by interference with NPC1 (Figure 4G). The mRNA expression levels of IFN-β, ISG15 and MX1 were significantly enhanced by PDCoV infection at 12 hpi. Moreover, the addition of siNPC1 to PDCoV-challenged cells significantly declined the expressions of IFN-β, ISG20 and MX1 (Figure 4H). These results demonstrated that active lysosomal function is beneficial for PDCoV and can be suppressed by 25HC.

Furthermore, 25HC inhibited the upregulation of TFEB, a transcription factor crucial for lysosomal biogenesis, induced by PDCoV infection, as shown by RT-qPCR and protein analysis (Figures 4I and J). Increased expression of ATGL, a lipase involved in lipolysis, was observed in PDCoV-affected cells at 4, 8 and 12 hpi (Figure 4J), indicating that lipolysis also participated in PDCoV-induced LD degradation.

### Nuclear translocation of TFEB is important for PDCoV-induced lipophagy

**Figure 5:**
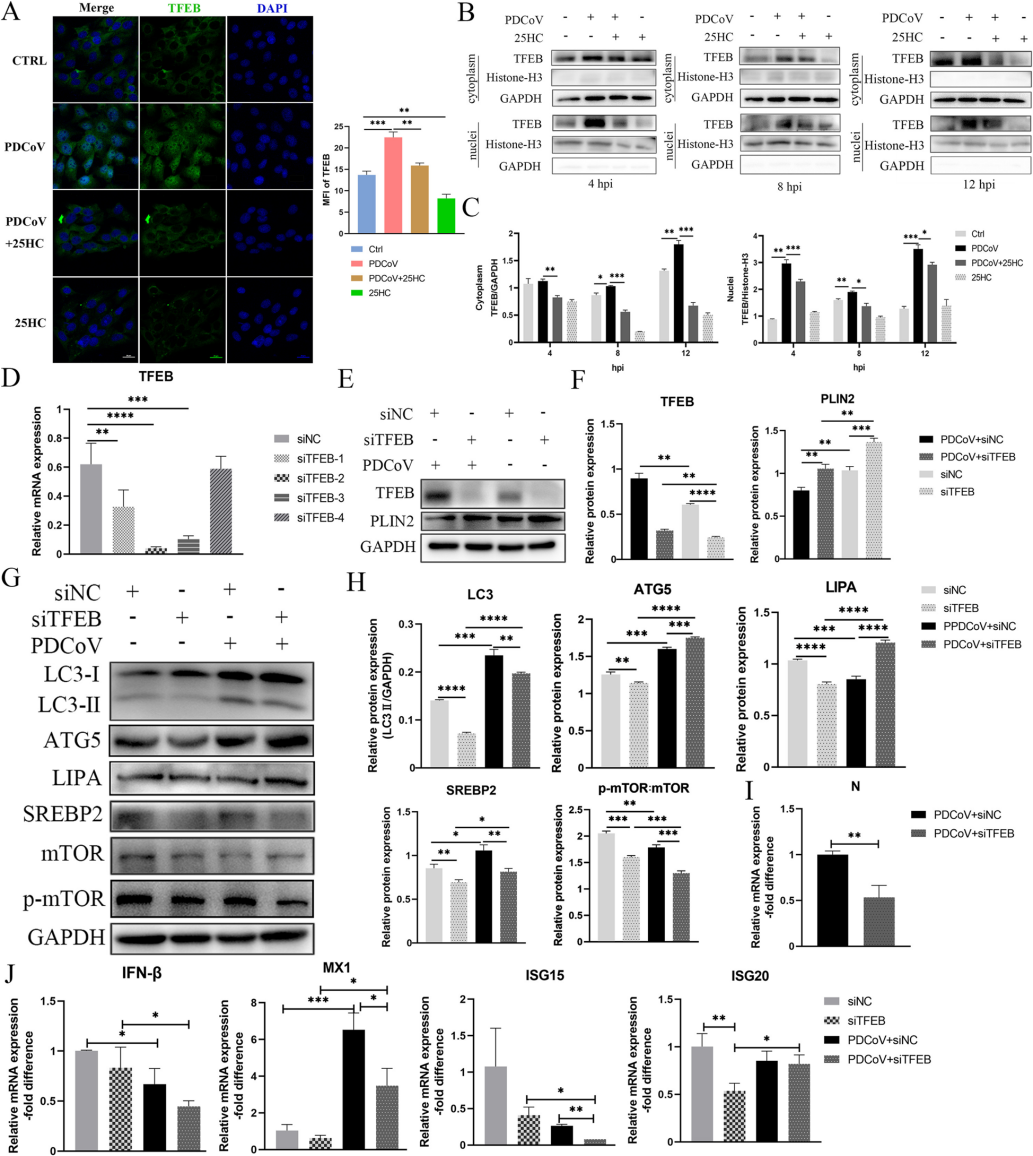
Nuclear translocation of TFEB is important for PDCoV-induced lipophagy. **A–C** LLC-PK1 cells were infected with PDCoV (MOI = 0.1) and treated with 50 μM 25HC for indicated time. **A** Cells were imaged by confocal microscopy using TFEB-specific antibody, TFEB was stained green and nuclei were stained blue with DAPI. Scale bar, 20 μm. Mean fluorescence intensity of TFEB were analyzed by Image J. **B** cytoplasm and nuclei extracts underwent western blot with TFEB-specific antibody. Histone-H3 and GAPDH were used as nuclear control and cytoplasmic control, respectively. **C** Immunoblot bands in panel B were quantified by Image J. **D** LLC-PK1 cells were transfected with four siRNAs targeting TFEB, the relative mRNA expression of TFEB was analyzed by qPCR. **E**–**J** LLC-PK1 cells were transfected with the second siRNA in panel D, followed by infection with PDCoV for 12 h. **E** and**G** The indicated protein expressions were analyzed by western blot, GAPDH was used as a loading control. **F** and **H** Bands in panel E and G were quantified by Image J. **I ** and **J** Relative mRNA expressions of indicated genes were analyzed by qPCR. Error bars, mean ± SD. *P < 0.05, **P < 0.01, ***P < 0.001.

Confocal fluorescence revealed significant TFEB translocation into nuclei following PDCoV infection, which was blocked by 25HC (Figure 5A). Next, the cytoplasmic and nuclear proteins were extracted at 4, 8 and 12 hpi. PDCoV infection markedly increased TFEB protein expression in the nucleus, whereas 25HC treatment decreased its nuclear expression and overall levels (Figures 5B and C).

To investigate the role of TFEB in regulating lipophagy, specific siRNAs targeting TFEB were constructed, and siTFEB-2 was applied in the following experiments (Figure 5D). Immunoblot analysis confirmed the effective knockdown of TFEB by siTFEB, which significantly increased the level of PLIN2 (Figures 5E and F). Besides, siTFEB obviously downregulated the expressions of LC3-II, ATG5, LIPA and SREBP2, whereas the knockdown of TFEB in PDCoV-affected cells significantly upregulated the expressions of ATG5 and LIPA. The activity of mTOR, indicated by the phosphorylation of mTOR, was inhibited after PDCoV infection and further decreased with the inhibition of TFEB (Figures 5G and H). Moreover, the inhibition of TFEB restricted the replication of PDCoV as indicated by reduced N gene expression (Figure 5I), whereas the downregulation of TFEB in the PDCoV group resulted in decreased IFN-β, MX1 and ISG15 compared with that in the PDCoV-infected cells after siNC transfection (Figure 5J). Overall, our results demonstrated that the translocation of TFEB is important for PDCoV infection and the expression of TFEB controls PDCoV-induced lipophagy.

### 25HC negatively regulates TFEB expression mediated by mTOR

**Figure 6.**
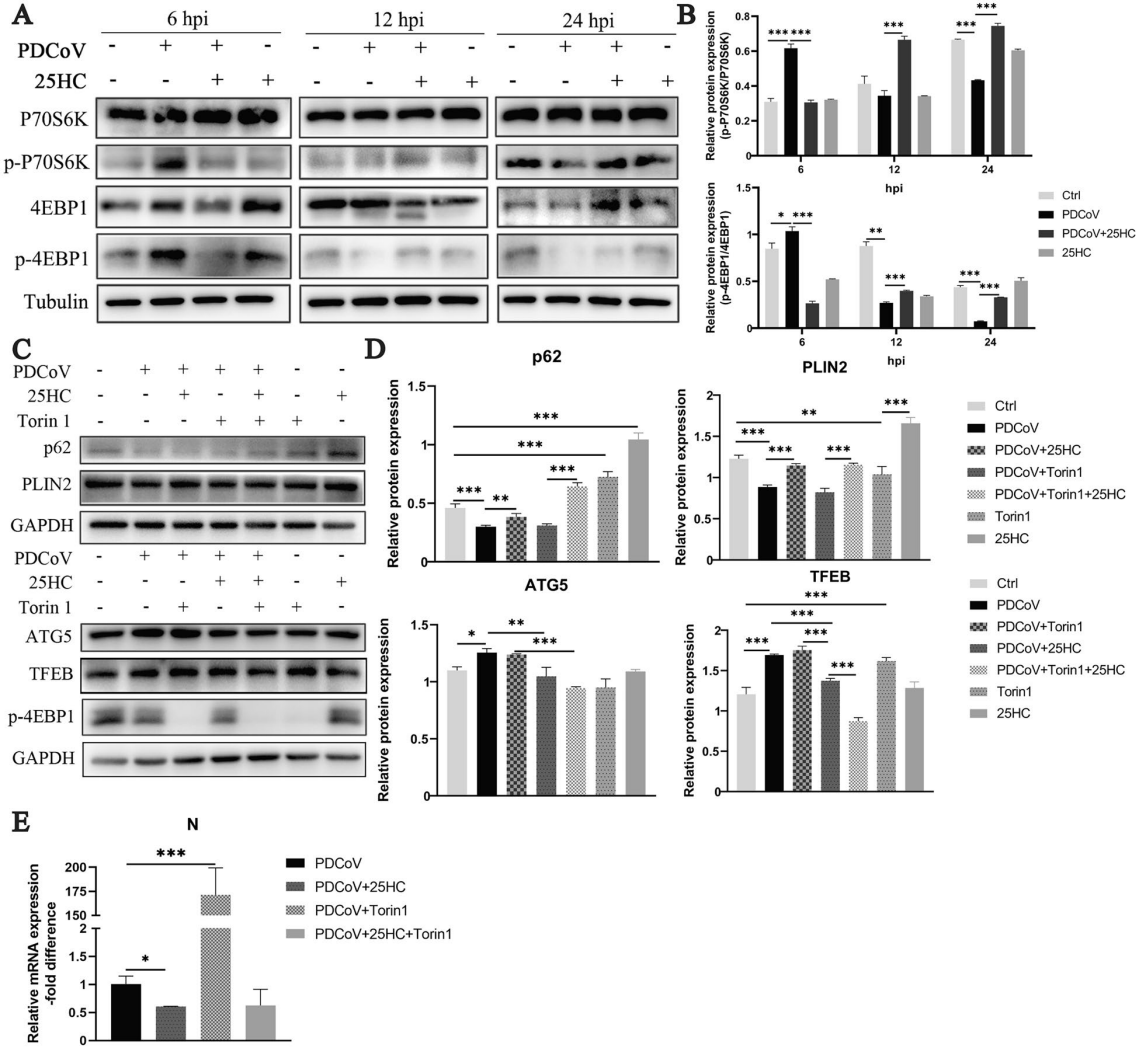
25HC negatively regulates TFEB expression mediated by mTOR. **A, B** LLC-PK1 cells were infected with PDCoV (MOI = 0.1) and treated with 50 μM 25HC for indicated time. **A** Indicated protein expressions were measured using western blot, with Tubulin being a loading control. **B** Immunoblot bands in panel A were quantified by Image J. **C–E** LLC-PK1 cells were infected with PDCoV (MOI = 0.1) and treated with 50 μM 25HC, 0.5 μM Torin1 for 12 h. **C** Indicated protein expressions were measured using western blot, GAPDH was used as a loading control. **D** Immunoblot bands in panel **C** were quantified by Image J. **E** Relative mRNA expression of PDCoV N was analyzed by qPCR. Error bars, mean ± SD. *P < 0.05, **P < 0.01, ***P < 0.001.

It is well established that mTOR serves as a nutrient sensor and that activated mTORC1 can inhibit the autophagy process [25]. To confirm the negative role of mTOR in PDCoV infection, we monitored dynamic mTORC1 activity by assessing the expressions of P70S6K, p-P70S6K, 4EBP1, p-4EBP1 at specific time points post infection. Our results revealed that mTORC1 was activated in the early infection stage at 6 hpi, evidenced by enhanced levels of p-P70S6K and p-4EBP1. However, with prolonged infection, mTORC1 activity significantly decreased at 12 and 24 hpi, 25HC reversed mTORC1 activity during PDCoV infection (Figure 6A and B). The eliminated p-4EBP1 expression confirmed the inhibitory effect of Torin1 on mTORC1. In addition, Torin1 increased ATG5 and TFEB expressions and decreased p62 expression in infected cells. Furthermore, when 25HC was administrated to mTOR-inhibited, PDCoV-infected cells, it suppressed the expressions of ATG5 and TFEB and restored the expressions of p62 and PLIN2, indicating inhibited lipophagy (Figures 6C and D). The expression of PDCoV N was subsequently analyzed in the presence of Torin1 to assess viral replication, Torin1 treatment significantly promoted PDCoV replication, which was also eliminated by 25HC (Figure 6E).

### 25HC inhibits PDCoV infection in a piglet model

**Figure 7.**
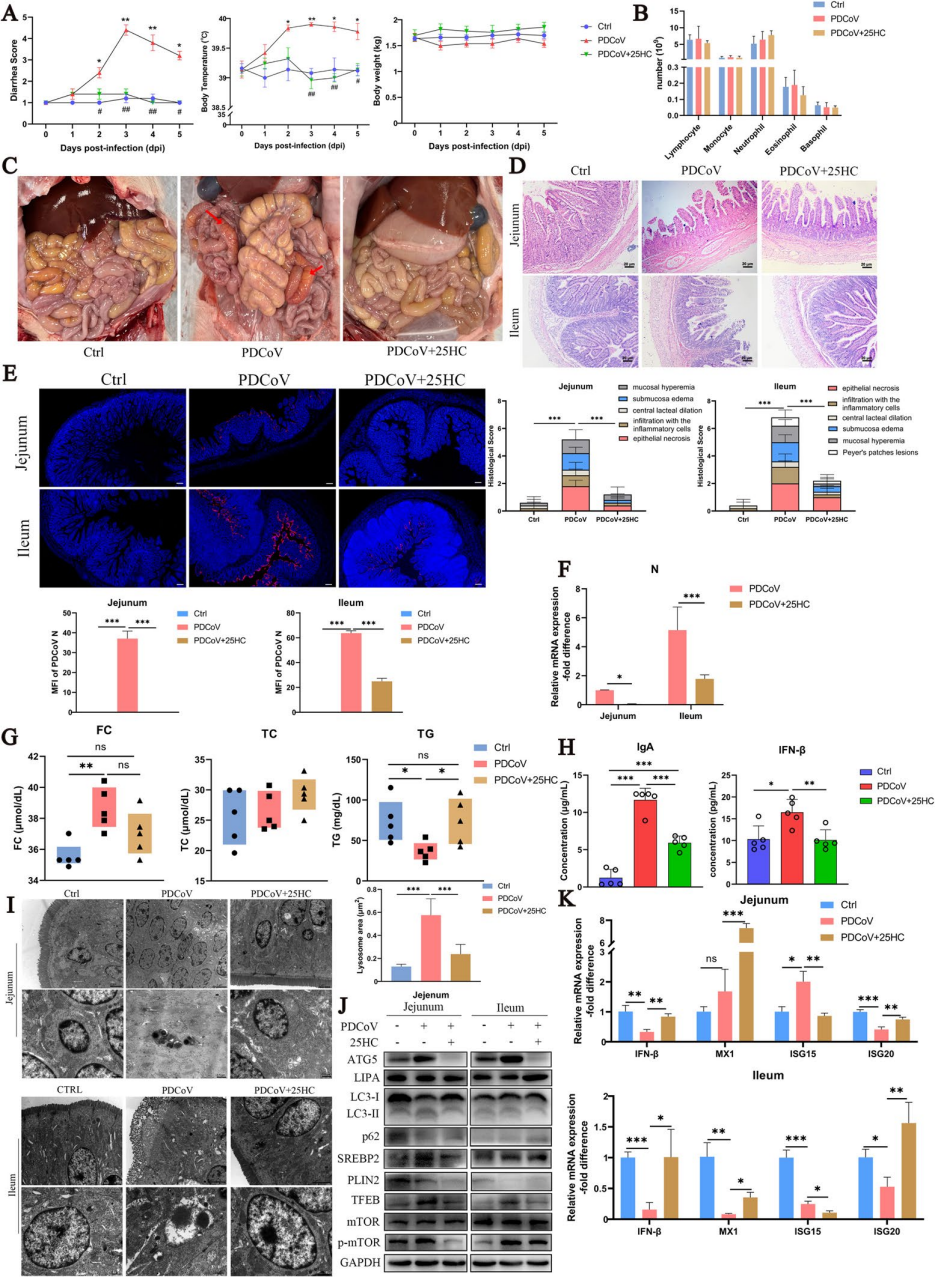
25HC inhibits PDCoV infection in a piglet model. 25HC (10 mg/kg) or vehicle (HβCD) was administered after PDCoV infection in piglets. **A** The diarrhea score, body temperature and body weight of piglets. **B** The number of leukocytes in blood. **C** The anatomical observation of piglets after they were sacrificed. The red arrows indicated the hyperemia in the intestines. **D** The H&E staining and histopathological score of Jejunum and ileum in piglets. Scale bar, 20 μm. **E** The PDCoV N protein was stained red using specific antibody and nuclei were stained blue with DAPI. Scale bar, 20 μm. The MFI of PDCoV N were analyzed by image J. **F** Relative mRNA expression of PDCoV N in jejunum and ileum were analyzed by qPCR. **G** The concentration of Free cholesterol (FC), Total cholesterol (TC) and triglyceride (TG) in serum were analyzed by commercial kits. **H** The porcine IgA and IFN-β content were measured by ELISA. **I** The ultrastructure of jejunum and ileum were observed using transmission electron microscopy. The scale bars were 2 μm and 0.5 μm, respectively. The area of lysosome was quantified using Fiji-image J. **J** The indicated protein expressions in jejunum and ileum were measured using western blot, GAPDH was used as a loading control. **K** The indicated gene expressions in jejunum and ileum were detected using qPCR. Error bars, mean ± SD. *P < 0.05, **P < 0.01, ***P < 0.001.

To further determine the effect of 25HC application in vivo, we employed a PDCoV-infected piglet model. Piglets in the PDCoV group exhibited aggravated diarrhea, increased body temperature, lethargy and decreased appetite at 3 dpi, while treatment with 25HC ameliorated these clinical symptoms (Figure 7A). No differences on the count of lymphocyte, monocyte, neutrophil, eosinophil and basophil were found among the three groups (Figure 7B). After the sacrifice, obvious hyperemia was observed in the small intestines of the PDCoV group, and no lesions were found in the control and 25HC treatment groups (Figure 7C). Histopathological examination revealed villus rupture, epithelial cell shedding, and hemorrhage in both the jejunum and ileum (Figure 7D). Replication of PDCoV in the jejunum and ileum was revealed by immunofluorescence, and treatment with 25HC almost removed the viral particles in the jejunum and markedly reduced the number of infected cells in the ileum, as verified by qPCR analysis (Figures 7E and F).

PDCoV infection led to elevated level of FC but reduced level of TG in the serum, and the administration of 25HC rose the TG content to the control level (Figure 7G). Interestingly, the levels of IgA and IFN-β in the serum were significantly upregulated in the PDCoV-challenged piglets compared with the control group, which was recovered by 25HC (Figure 7H). Furthermore, transmission electron microscopy images showed numerous abnormal nuclei and accumulated lysosomes in the jejunum, meanwhile destroyed villi and unreleased viral particles were observed in the ileum. The application of 25HC repaired the pathological ultrastructure in the jejunum and ileum (Figure 7I).

PDCoV infection induced autophagy in the jejunum and ileum, as indicated by enhanced expressions of ATG5, LC3-II and reduced expression of p62 and LIPA, all of which were recovered by 25HC treatment. Additionally, PDCoV infection increased expressions of SREBP2, TFEB, and p-mTOR, while reducing PLIN2 expression in the jejunum and ileum. The abnormal expressions of SREBP2, TFEB, and p-mTOR in infected piglets were all reversed by 25HC (Figure7J). In addition, the expressions of IFN-β and ISG20 were significantly decreased in the jejunum and ileum after PDCoV infection, whereas the expression of ISG15 was upregulated in the jejunum and the expressions of MX1 and ISG15 were decreased in the ileum. The administration of 25HC promoted the expression of IFN-β, MX1 and ISG20 in both the jejunum and ileum but suppressed the expression of ISG15 (Figure 7K).

## Discussion

Viruses utilize host cholesterol for entry and replication. The dynamic processes of cholesterol, such as cholesterol metabolism and trafficking, play critical roles in the viral lifecycle [26]. The LD, a dynamic cellular organelle that stores neutral lipids, is crucial for cholesterol recycling, and the regulation of LD homeostasis and LD-related proteins were shown to function in innate immunity [27]. A previous study reported that LDs accumulate early in PDCoV infection, and the data in the present study indicate a decrease in LD content and neutral lipids as infection progresses (Figure 1). LDs exhibit dual effects during viral infections: in addition to the beneficial effects of LDs on viral replication through the construction of compartments or providing energy [28–30], the antiviral effects of LDs were also discovered recently. In the current study, the induction of LDs by oleic acid (OA) impeded the replication of PDCoV, however, the low concentration of OA treatment did not increase PLIN2 expression in normal cells because of the simultaneous induction of LD synthesis and degradation. Moreover, we observed increased expression of autophagy markers and increased LIPA expression during PDCoV infection in the presence of OA (Figure 2). Given the augmented cholesterol production, we hypothesize that PDCoV may utilize lipophagy to reduce LDs. Given that LD accumulation is required for interferon response in the early infection of virus [12], we measured two ISGs, ISG15 and MX1, which can be induced by type I interferon and possess antiviral capacities. The results showed that accumulated LDs by combination of OA and Baf A1 dramatically increased the expression of ISG15 and MX1.

Autophagy is beneficial for the host to recognize and eliminate pathogens including viruses and can also be converted to facilitate viral replication [31]. We have shown that autophagy flux is constantly active during PDCoV infection. To underscore the importance of lipophagy in PDCoV-infected cells, we observed a further reduction in PLIN2 levels upon treatment with EBSS (Figure 3). Lipophagy can also be manipulated by DENV to release free fatty acids and generate ATP, which is required for efficient viral reproduction [32]. The importance of lipophagy for the degradation of LDs is same to lipolysis, interestingly, to some extent, part of lipids released by lipophagy are esterified in a short time to supplement the pool of LDs [33]. Central to LD breakdown is lysosomal activity, which is critical for maintaining cholesterol homeostasis by hydrolyzing LDs. Various viruses have evolved to interfere with autophagosome maturation and fusion with lysosomes to evade degradation. Kaposi’s sarcoma-associated herpesvirus (KSHV) has been shown to block the fusion of autophagosomes with lysosomes by promoting the Rubicon–Beclin1 interaction and inhibiting the activity of VPS34 [34]. The HBV X protein is shown to stimulate autophagy initiation and impede lysosomal acidification [35]. However, the nexus between autophagosomes and lysosomes is yet well-studied in PDCoV infection. Our findings indicate enhanced lysosomal function and upregulated NPC1 expression during PDCoV infection. Of interest, inhibition of NPC1 with U18666A promotes PDCoV replication, which might be due to its blockage of esterification [24]. Deficiency of NPC1 leads to type-C form of Niemann-Pick (NPC) disease along with accumulated cholesterol in lysosomes [36]. NPC1 is also recognized as a crucial receptor for the Ebola virus and Filovirus [37, 38]. Silencing of NPC1 supports its positive role in PDCoV infection (Figure 4), highlighting its importance in facilitating viral cholesterol utilization. Besides, PDCoV infection triggers nuclear translocation of TFEB, and TFEB knockdown impedes PDCoV replication and lipophagy (Figure 5). These results indicate the comprehensive activation of lysosomes during PDCoV infection. The dephosphorylation and nuclear translocation of TFEB in HIV-1-infected macrophages stimulate autophagy and lysosomal biosynthesis, moreover, the sequestration of TFEB by HIV-1 Nef inhibits the maturation of autophagosomes [39]. Furthermore, the autophagy factor IRGM elicits lysosomal activation controlled by TFEB during HIV infection [40]. Thus, active lipophagy and lysosome function controlled by TFEB probably play pivotal roles in PDCoV replication and cholesterol recycling.

Previous studies have reported the complex relationship between 25HC and autophagy. 25HC has been shown to inhibit autophagy in a non-small cell lung cancer model [41], moreover, its inhibitory effect is implied to be connected with the induction of reactive oxygen stress [42]. We hypothesized that the blockade of autophagy by 25HC is beneficial for the host. In this study, we identified 25HC as an inhibitor of lipophagy and lysosomal activation, which consequently promotes lipid droplet (LD) accumulation. The regulatory effect of mTORC1 on autophagy is well established, simply, mTORC1 inhibits autophagy maturation and autophagosome-lysosome fusion [43]. Pieces of evidence have demonstrated that in the interphase cells, mTORC1 inhibition unlocks autophagy flux and facilitates the nuclear translocation of TFEB [44, 45]. In addition, lysosome possesses the ability to affect autophagy induction and mTORC1 signaling [46]. However, the role of mTORC1 in lipophagy is yet to be well elucidated. In the present study, we confirmed that mTORC1 inhibition turns on the lipophagy and permits the activation of TFEB. Reduced lysosomal function and TFEB activation restrain PDCoV replication, which is further promoted by the Torin1 treatment. Interestingly, the activated autophagy flux, lysosomal function and inhibited mTORC1 activity induced by PDCoV are all reversed by 25HC. In addition, enhanced mTORC1 in the early infection stage is also restored by 25HC.

In the piglet model, PDCoV infection predominantly induced lesions in the jejunum and ileum, which is consistent with previous findings [47]. Interestingly, abundant viral particles were found in the ileum and the number of PDCoV-positive cells in the ileum seemed greater than the number in the jejunum, indicating that the ileum might be the main invasive site of PDCoV. Similar to the in vitro results, the application of 25HC inhibited the PDCoV infection in vivo and suppressed the autophagy flux. However, PDCoV enhanced mTORC1 activities in the jejunum and ileum, contrasting its effect in vitro. It is known that mTORC1 activity is tightly related to protein synthesis, which is the most energy-intensive and nutrition-intensive process and needs a large portion of cellular resources [48]. We speculate that PDCoV inhibits mTORC1 in cells due to limited energy, whereas tissues provide ample resources supporting mTORC1 activation during viral replication. It is intriguing that the 25HC application restored the active mTORC1 expression following PDCoV infection, demonstrating its separate regulatory effects on autophagy and mTORC1 in vivo. Serum IgA and IFN-β levels reflect the overall immune response against PDCoV, potentially preventing viremia. Nevertheless, the overall downregulated expression of ISGs seems to facilitate the replication and invasion of PDCoV in the jejunum and ileum, whereas stimulation of ISGs by 25HC could aid in viral clearance.

**Figure 8:**
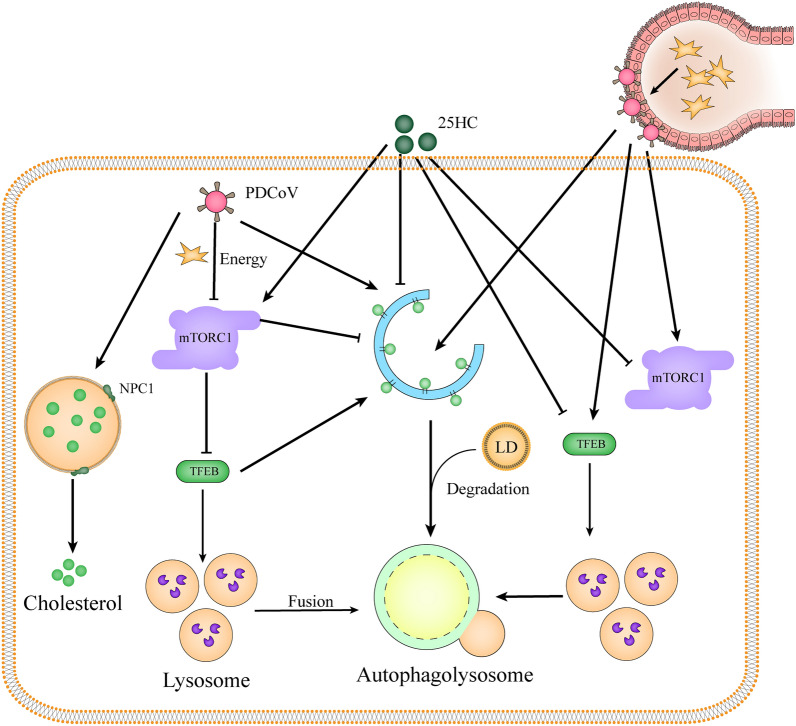
**The mechanism schematic diagram of 25HC inhibiting PDCoV replication in vitro and in vivo.**

In summary, our findings provide compelling evidence that 25HC modulates lipophagy and mTORC1 activity, leading to LD accumulation and reduced cholesterol recycling (Figure 8). However, the conditions under which PDCoV switches on or off the mTORC1 activity and the potential effect of TFEB on cholesterol metabolism during PDCoV infection still need to be explored.

## Data Availability

All data generated or analysed during this study are included in this published article.

## Abbreviations

25HC: 25-Hydroxycholesterol
BafA1: bafilomycin A1
EBSS: Earle’s balanced salt solution
FC: free cholesterol
IFN-β: interferon-β
ISG: interferon-stimulated gene
LC3-II: microtubule-associated protein light chain 3-II
LD: lipid droplet
OA: oleic acid
PDCoV: porcine deltacoronavirus
TFEB: transcription factor EB
TG: triglyceride
